# All optical tunable RF filter using elemental antimony

**DOI:** 10.1515/nanoph-2023-0654

**Published:** 2024-01-26

**Authors:** Samarth Aggarwal, Nikolaos Farmakidis, Bowei Dong, June Sang Lee, Mengyun Wang, Zhiyun Xu, Harish Bhaskaran

**Affiliations:** Department of Materials, University of Oxford, Parks Road, Oxford OX1 3PH, UK

**Keywords:** microwave photonics, all-optical filter, low-pass filter, thin-film antimony

## Abstract

In the past decade, the proliferation of modern telecommunication technologies, including 5G, and the widespread adoption of the Internet-of-things (IoT) have led to an unprecedented surge in data generation and transmission. This surge has created an escalating demand for advanced signal processing capabilities. Microwave photonic (MWP) processors offer a promising solution to satisfy this unprecedented demand for data processing by capitalising on the high bandwidth and low latency achievable by optical systems. In this work, we introduce an integrated MWP processing unit for all-optical RF filtering using elemental antimony. We exploit the crystallisation dynamics of antimony to demonstrate a photonic leaky integrator, which is configured to operate as a first-order low-pass filter with a bandwidth of 300 kHz and ultra-compact footprint of 16 × 16 μm^2^. We experimentally demonstrate the implementation of such a filter as an envelope detector to demodulate an amplitude-modulated signal. Finally, a discussion on achieving bandwidth tunability is presented.

## 1 Introduction

Microwave photonic (MWP) processors offer inherent benefits offered by photonics such as insensitivity to electromagnetic interference [1]. So far, there has been significant interest in using MWP processors for filtering operations in the optical domain [2]–[7]. Such MWP processors can be embedded with optical sensors in IoT-based devices to pre-process real-time data. This would reduce the computational load while avoiding the losses associated with electro-optic conversions in the processing unit. Various MWP processors have been demonstrated as all-optical delay lines [8], [9] and for filtering operations such as notch filters [10]. Most implementations of MWP processors so far have utilized coherent light sources and often use resonator-coupled Mach–Zehnder interferometer (MZI) devices [11]–[14], photonic crystals [9] or are based on nonlinear effects such as stimulated Brillouin scattering (SBS) [15]–[19]. MWP filters using ring resonators and interferometers suffer from large device footprints and have a periodic transfer function limited by their free spectral range [19], ultimately limiting the tuning bandwidth. For SBS-based MPWs, while low-power integrated solutions have been proposed [16], using chalcogenide waveguides makes them incompatible with the CMOS process, thus limiting scalability and adoption.

While current integrated solutions can achieve satisfactory tunability and high bandwidth [20], [21], they are still limited by their large footprints and incompatibility with foundry processes. To achieve adoption of such MWP processors, there have been efforts to reduce their overall device footprint and energy budget, mainly using integrated photonic solutions [22], [23]. The increasing maturity of integrated photonic platforms has allowed for co-integration of key components such as lasers and modulators [4], [24], [3].

This work introduces an ultra-compact MWP filter based on elemental antimony. This material was recently demonstrated by the authors (not all authors were involved in the previous work) to function as a dynamic phase change material (PCM) [25], [26]. These properties were explored for photonic phase change memory and optical weighting [25], [26]. Such antimony-based memory has been shown to have a tuneable, volatile behaviour in milliseconds to seconds, depending on the device geometry. This volatility has been attributed to the spontaneous recrystallisation of the amorphous phase. In previous work, a thickness-dependent volatile behaviour for thin film antimony has been demonstrated [25]–[28]. Here, we exploit the material-level properties, specifically the spontaneous recrystallisation of antimony as a leaky integrator and demonstrate a first-order low pass filter with a bandwidth of 300 kHz (3 dB).

In Table 1 we summarise various MWP filters techniques reported in literature. It is worth noting that our current proof of concept work is limited to a narrow band low pass filter. Nevertheless the bandwidth of such a system can further be increased by designing better photonic circuits, integrating functional materials and resonator based MWP designs. The purpose of this work is to introduce MWP based on elemental antimony. Our implementation can also be readily adapted for use with phase change materials demonstrating multilevel memory operation and tuneable volatility such as Ge_2_Se_2_Te_5_ [29], Ge_3_Sb_2_Te_6_ [30] and VO_2_ [31]_,_ amongst others.

**Table 1:** Summary of various MWP filters technologies.

Technique	Filter type	Bandwidth (3 dB)	Footprint	Platform	Reference
Photonic crystal- delay line	Notch, bandpass	50 GHz	1.5 mm long	GaInP/GaAs	[9]
MZI-ring resonator	Notch	635 MHz	1.75 mm^2^	Si	[7]
Ring-MZI	Notch, bandpass	1–2 GHz	6 × 4 mm^2^	InP	[3]
Ring resonator	Notch	247–840 MHz,	8.73 mm long	SiN	[12]
SBS	Stop-pass	32–88 MHz	6.5-cm-long	As_2_S_3_	[16]
SBS	Notch filter	126 MHz	6.5-cm long	As_2_S_3_	[18]
PCM	Low-pass filter	300 kHz	16 × 16 μm^2^	Si	This work

MZI, Mach Zender interferometer; SBS, stimulated Brillouin scattering; PCM, phase change material.

## 2 Results

### 2.1 Filter design

**Figure 1:**
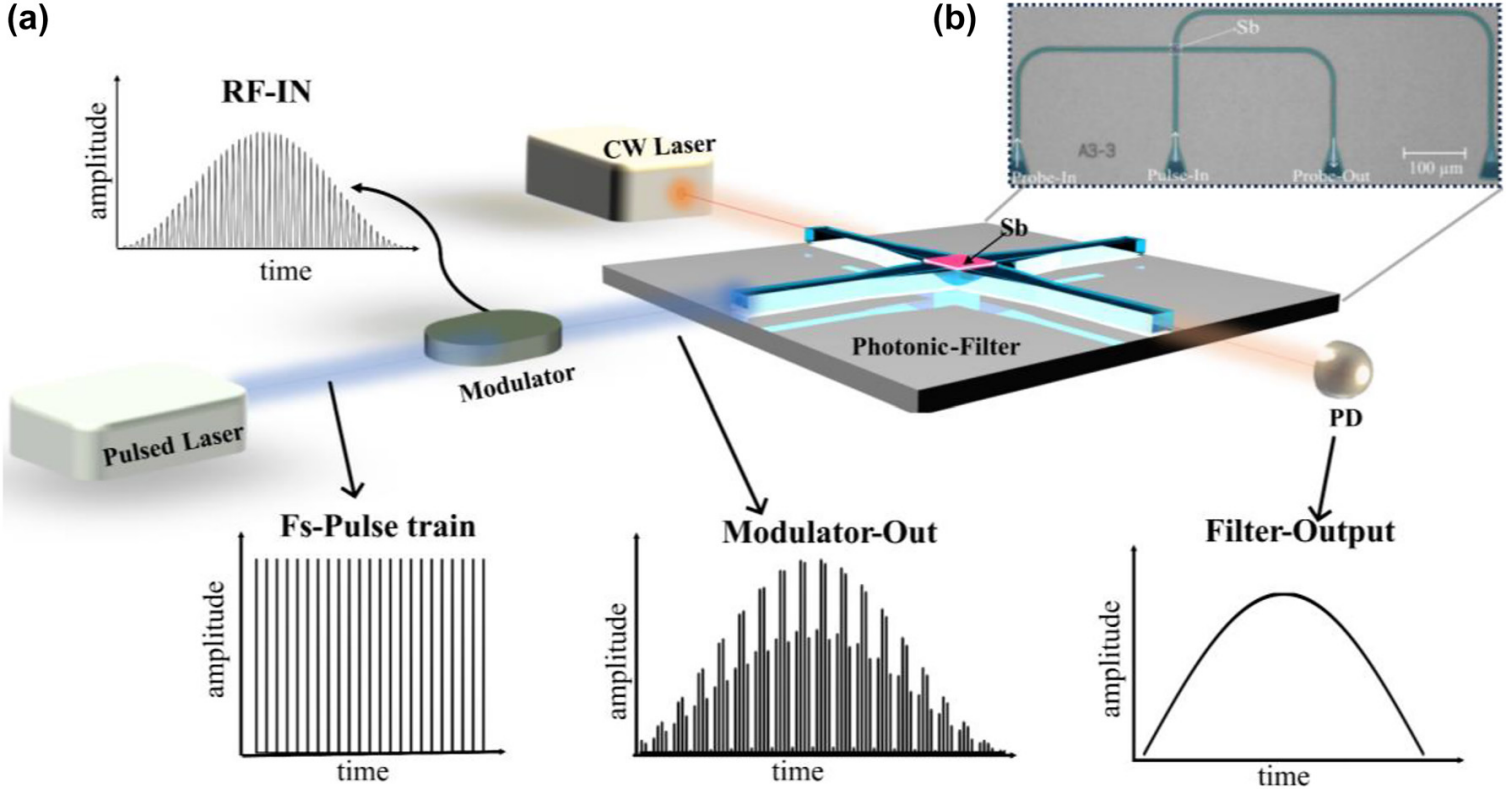
Antimony based microwave photonic filter. (a) Schematic of Sb-PCM based photonic filter design. An arbitrary rf signal is discretised and amplitude modulated on a fs pulse series. These modulated fs pulses (modulator-out) are then used to amorphize Sb. A CW laser is used to observe the change in transmission (filter-out). (b) False coloured optical image of the fabricated photonic device consisting of a waveguide crossing with 5 nm Sb sputtered on top and grating couplers to couple light to perform filter operation.

The concept of a microwave photonic filter is demonstrated in Figure 1(a). The design consists of a photonic waveguide crossing with a thin film of antimony (Sb) on the waveguide at the crossing. As demonstrated in previous work [25], [26], femtosecond (fs) pulses can result in amorphisation of Sb. Crystalline and amorphous antimony have different optical absorption coefficient (*k* = 2.81 for crystalline and *k* = 0.61 for amorphous phase at 1550 nm) [26], thus resulting in a change in the transmission state of the photonic device, from low transmission to higher transmission. Different transmission states are achieved depending on the amplitude of the amorphisation pulses.

In our implementation of a photonic RF filter, an arbitrary RF input signal is discretised, and amplitude modulated on a fs pulse series (Fs-pulse train) using an optical modulator as shown in Figure 1(a). Here the pulse repletion rate determines the sampling rate of the RF input signal. The modulated fs pulses (modulator-out) are then used to switch antimony. A continuous wave (CW) laser is used in the probe line, and a photodetector records the transmission change (filter-output). In our experiments, a fs pulse with a repetition rate of 3.3 MHz and pulse width of 800 fs is used.

In Figure 1(b), we show a false-coloured optical microscope image of the photonic device used for implementing an integrated photonic RF filter. The device consists of a 16 × 16 μm^2^ waveguide crossing, with grating couplers to couple light in and out of the device. The device consists of a 2 × 2 μm^2^ of 5 nm-thick ‘active’ material, i.e., Sb thin film. The device is based on a standard 220 nm silicon on insulator (SOI) photonic platform. A half-etched, single-mode ridge waveguide for operation at 1550 nm wavelength is fabricated via electron beam lithography.

### 2.2 Leaky integrator

**Figure 2:**
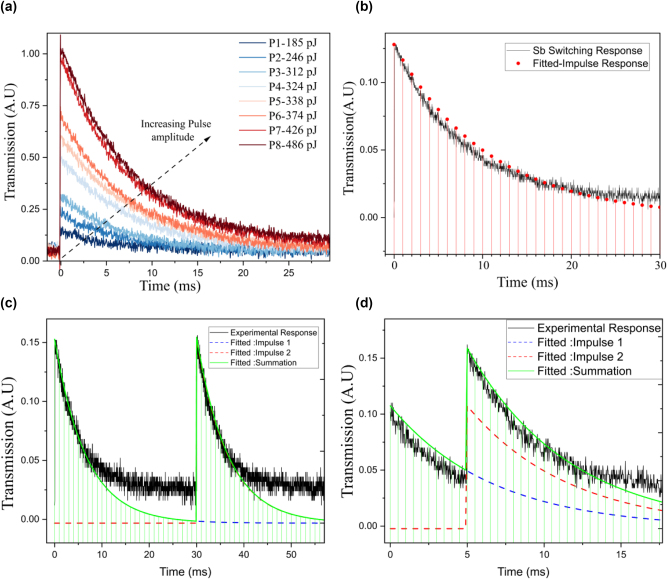
Switching response of antimony device. (a) Volatile switching of antimony with varying pulse energies from P1–P8 (185 pJ, 246 pJ, 312 pJ, 324 pJ, 338 pJ, 374 pJ, 426 pJ, 486 pJ, respectively). (b) Impulse response of a leaky integrator is fitted with the switching response for the Sb filter device. (c–d) Superposition of impulse response for Sb device. Experimentally obtained response (in black) is fitted to simulated responses (in blue and red). The summation of two simulated responses (in green) matches well with the experimentally obtained response for ‘long’ separation of 30 ms and a ‘short’ duration of 5 ms, respectively.

First, we investigate the switching response of our device using fs pulses by recording the change in transmission using a low-power CW probe laser. The response to single fs pulses of increasing pulse energy from P1 to P8 (185 pJ–486 pJ), is recorded in Figure 2(a). Starting with the low transmission crystalline phase, high energy single fs pulse leads to amorphisation, thus increasing the transmission. As determined in earlier work [25], the amorphisation time using femtosecond pulse is 2 ns. Due to the spontaneous recrystallisation of the amorphous phase, a volatile change in transmission is observed. This volatile switching response of Sb has a response equivalent to the impulse response of a leaky integrator.

For a stream of sampled data, a leaky integrator adds up the input data while ‘forgetting’ some of the previous data, i.e. ‘leaking’ it. A leaky integrator can thus be used to average a data stream, effectively functioning like a low-pass filter and is represented by the following equation:

$$y\left[n\right]=\alpha x\left[n\right]+\left(1-\alpha \right)y\left[n-1\right],\;where\;0\leq \alpha \leq 1$$

where *x*[*n*] is the *n*th data sample, and alpha (*α*) is the leaky parameter. Thus, for *α* = 1, no averaging takes place and reducing *α* results in stronger averaging. This can be further analysed by taking the *z*-transform to get the following:

$$Y\left(z\right)=\alpha X\left(z\right)+\left(1-\alpha \right)Y\left(z\right)z^{-1}$$

This can then be rearranged to get the transfer function *H*(*z*):

$$H\left(z\right)=\frac{Y\left(z\right)}{X\left(z\right)}=\frac{\alpha }{1-\left(1-\alpha \right)z^{-1}}$$

Thus, the impulse response of a leaky integrator can be written as $h\left[n\right]=\alpha {\left(1-\alpha \right)}^{n}$ . The single pulse response of the antimony filter device is thus fitted with the impulse response trend of a leaky integrator in Figure 2(b) and shows good agreement. Here the red dot represents the impulse response *h*[*n*] of a leaky integrator at different time steps ‘*n*’.

Next, we explore the superposition properties of the device. For a linear filter with response *H*(*s*) and stimulus *s*_1_ and *s*_2_, $H\left(s_{1}+s_{2}\right)=H\left(s_{1}\right)+H\left(s_{2}\right)$ , i.e., the response of two stimuli performs the summation of individual responses in the hardware. Therefore, two pulses separated in time are sent through the device. In the first case, the two pulses are separated by 30 ms, as shown in Figure 2(c). The second pulse is sent a long time after the first one. The impulse responses of a leaky integrator for two pulses, separated by 30 ms, are fitted and shown as dotted lines in blue and red, and the net expected change is shown in green. The response of our device (in black) matches well with the impulse response of a leaky integrator.

In the second case, the two pulses are separated by a ‘short’ duration of 5 ms. As before, the impulse responses of 2 pulses separated by 5 ms are plotted as dotted lines in blue and red in Figure 2(d). The summation of the two impulse responses is shown in green. The impulse response of a leaky integrator fits well with the transmission response of the Sb filter device, thus showing the linearity of stimulus responses.

It is worth noting that the switching and the recrystallisation dynamics of a PCM-based device is a complex function of the pulse amplitude, number of pulses and the transmission state of the device. While working with many pulses, the recrystallisation dynamics of the material can be enhanced due formation of a large number of subcritical nuclei [32] during melt-quench. Moreover, switching pulses are absorbed weakly while in a higher transmission state, resulting in saturation of the achievable transmission level. We use this richness present in our device to demonstrate a low-pass filter, and the exact nature of this phenomenon will require further studies.

### 2.3 Frequency response

Any input signal can be discretised in time and represented as impulses with weighted amplitude and shifted in time. Thus, the response of such input signals through the Sb filter device can be predicted easily as the sum of the impulse response of Sb filter cells weighted with the impulse amplitude. Another consequence of this property is that the output at any time ‘*t*’ is the weighted average of all the previous impulse responses. This results in the smoothing of the input signal, or in other words, performs a low-pass filter operation on the input data.

Next, we test this property of a low-pass filter for our device. As described previously, while keeping the pulse repetition rate fixed at 3.3 MHz, rectified sine waves of varying frequency are modulated onto a train of femtosecond pulses. The transmission response of the Sb device for various input signals is reported in Figure S3. It is observed that increasing the input signal frequency decreases the amplitude of the output sine wave signal, thus acting as a low-pass filter. When Sb is switched to amorphous phase the transmission of our device increases initially, but subsequently drops due to spontaneous recrystallisation. This results in jitter in the device response. This jitter noise can further be reduced by increasing the sampling rate.

**Figure 3:**
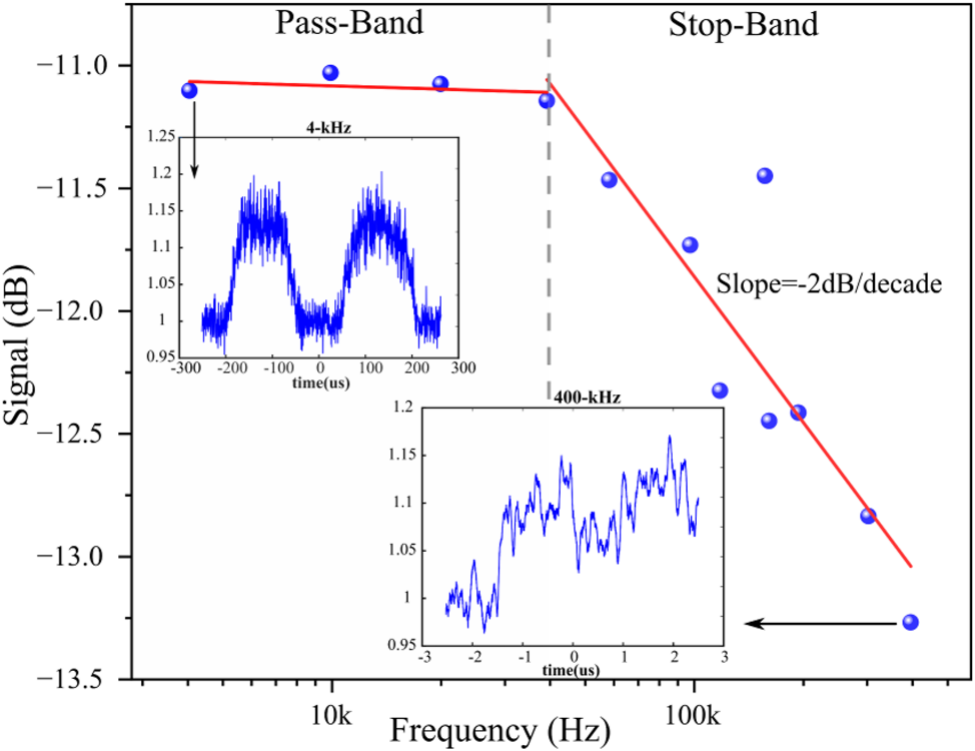
Frequency response analysis of sinusoidal data (details in Figure S3) is performed to determine the pass-band and stop band of the filter. Inset shows the experimentally obtained sine wave response of the device for a low frequency (4 kHz) and high frequency (400 kHz) input signal.

Next, the analysis for individual frequency response is performed by taking the fast Fourier transformation (FFT) of the output response. The amplitude (in dB) of the device output is plotted for various frequencies in Figure 3. This plot determines the pass band and the stop band for the Sb device filter. A bandwidth of 300 kHz (at 3 dB loss) with an attenuation of −2 dB/decade is obtained for an integrated photonic RF filter.

### 2.4 Envelope detector

An envelope detector takes in a high-frequency amplitude-modulated (AM) signal as input and returns the envelope of this signal, which is the low-pass output. Therefore, to demonstrate the performance of the Sb device as an envelope detector, a 4 kHz sine wave signal is AM with different carrier frequencies ranging from 40 kHz to 2 MHz.

**Figure 4:**
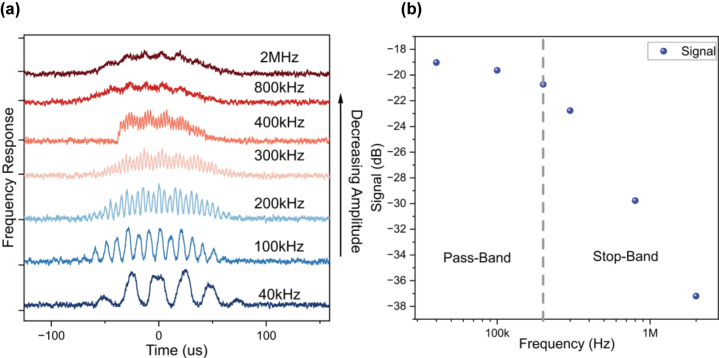
Demonstration of low pass filtering operation.(a) A 4 kHz signal is amplitude modulated with various carrier frequencies, as reported in the graph. Increasing the carrier frequency results in the suppression of the amplitude of the carrier signal. (b) Frequency response analysis of the sine wave of data shown in (a) is performed to determine the pass-band and stop-band of the filter.

As determined above, the Sb device has a 3 dB bandwidth of 300 kHz. Thus, frequencies above this range are filtered from the input signal. The response of the Sb device for various carrier frequencies is shown in Figure 4(a). As expected, frequencies >300 kHz are filtered from the 4 kHz sine wave signal, observed as a decrease in the carrier signal amplitude, thus showing excellent performance as an envelope detector. To further analyse the performance of the envelope detector, as before, the amplitude of the carrier signal wave is plotted for various frequencies in Figure 4(b). An exponential suppression of the high-frequency signal is observed, thus demonstrating the excellent performance of the Sb device as an envelope detector.

## 3 Tuneable filter

So far, the Sb-based photonic device has been shown to function as a low-pass filter of fixed bandwidth. The frequency response depends on the leaky parameter of the filter. A ‘leaky’ filter would result in a higher cutoff frequency. For the Sb device, the degree of leakiness depends on the retention time of Sb. As demonstrated in previous works, the volatility of Sb can be tuned by varying the probing power [25] and the thickness of the thin film [26], [27].

**Figure 5:**
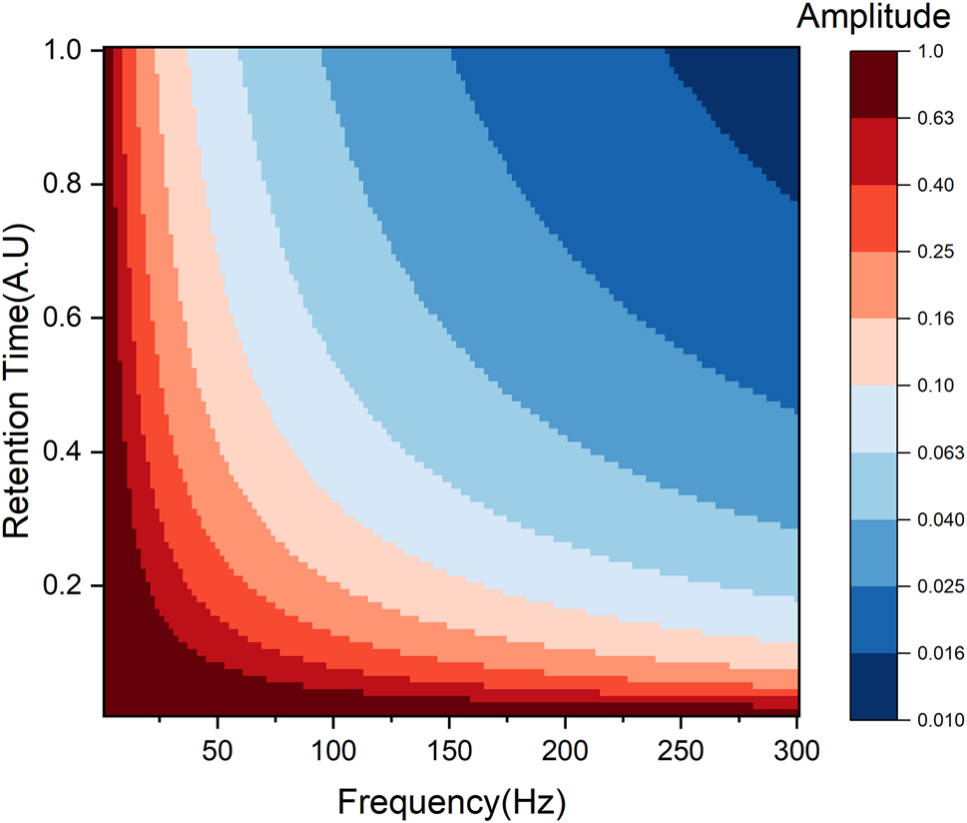
Simulation results demonstrating the effect of retention time of Sb on the frequency cutoff for low-pass filtering.

Therefore, to evaluate the effect of tuning the Sb device’s retention time (volatility) on the input sine wave signal, we simulate the response function for various frequencies in Figure 5. Using fast Fourier transformation, the amplitude of the output response is determined. Increasing the retention time results in a decrease in the low-pass cutoff frequency. Thus, by varying the volatility of the device, a tuneable low-pass filter can be achieved. This tunability can be exploited to achieve complex filtering functions and filter banks using an array of waveguides with a varying film thickness of Sb.

## 4 Conclusions

We have demonstrated elemental Sb-based photonics as an integrated MPW processor configured as an optical low-pass filter. We exploit the intrinsic recrystallisation dynamics of the material-system to demonstrate a low-pass filter. The low-pass filter has a 3 dB cutoff bandwidth of 300 kHz and a stop band with an attenuation of −2 dB/decade. We demonstrate an application of this low-pass filter as an envelope detector to demodulate amplitude-modulated signals. We achieve this using an ultracompact device with a footprint of 16 × 16 μm^2^ with an active area of only 2 × 2 μm^2^. The small footprint and the ease of fabrication of the device allow for the co-integration of CMOS electronics. While our current demonstration has a fixed bandwidth, we provide discussions for achieving the tuneability of bandwidth by tuning the recrystallisation dynamics by changing the probe power. Such tunability opens up the use of volatile antimony for many applications in signal processing and data encryption.

## 5 Methods

### 5.1 Device fabrication

The waveguides are fabricated on a standard 220 nm SOI wafer. The photonic device consisting of the waveguides and the waveguide crossing is patterned using a positive electron beam-sensitive photoresist (AR-P 6200). The waveguides are half etched using reactive ion etching. Using a subsequent lithography process, the windows for Sb deposition are defined. The thin film of antimony is then deposited on the waveguide crossing using an RF sputtering system at a 3.3 nm/min deposition rate in an argon environment using a sputtering target from Testbourne.

### 5.2 Experimental setup

The experimental setup illustrated in Figure S1 consists of a pump and probe setup as used before, with additional pump modulation electronic circuits described in section S1. A fs laser from Pritel (FFL-TW) is used to switch Sb, and a CW laser (Santec, TSL-550) is used to monitor the transmission change. To achieve the modulation of RF signal-input onto the fs laser pulses, an acoustic optical modulator (AOM) from G&H (Fiber-Q 1550 nm) is used. This results in the discretisation of the RF signal with each sample represented as $X\left[n_{i}\right]$ . The amplitude modulated fs pulses are amplified using an erbium doped fibre amplifier (EDFA). Using a CW probe laser, the transmission of the device is monitored using a 125 MHz photodetector from Newport (1811) and recorded onto a 4 GHz oscilloscope.

## Supplementary Material

Supplementary Material Details
