# Supplementary material for: All optical tunable RF filter using elemental antimony
Source: Nanophotonics. 2024 Jan 26;13(12):2223–9. doi: 10.1515/nanoph-2023-0654 (PMC11501610; doi:10.1515/nanoph-2023-0654)
Supplement: Supplementary file 1 — Supplementary Material Details [file j_nanoph-2023-0654_suppl_001.docx]

**Supplementary information for: All optical RF filter using elemental antimony**

Samarth Aggarwal^1^, Nikolaos Farmakidis^1^, Bowei Dong^1^, June Sang Lee^1^, Mengyun Wang^1^, Zhiyun Xu^1^ and Harish Bhaskaran*^1^

^1^Department of Materials, University of Oxford, Parks Road, Oxford OX1 3PH, UK

*Corresponding author: E-mail: [harish.bhaskaran@materials.ox.ac.uk](mailto:harish.bhaskaran@materials.ox.ac.uk)

# Experimental Setup


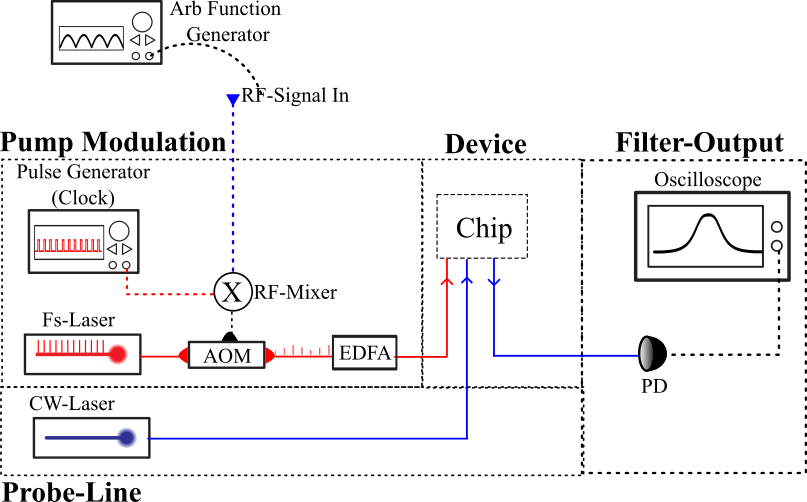


Figure S 1: Schematic of the experimental setup consisting of a pump line and a probe line. Femtosecond pulses are modulated with RF signal and used to switch the device. The change inn transmission is record using a CW laser and recorded on a photodetector on an oscilloscope.

The femtosecond pulse laser used in the experiments has a frequency of 40 MHz, resulting in a sampling frequency of 40 MHz, which is too high for the Sb filter device. A clock divider circuit is needed to reduce the sampling frequency. Therefore, to simplify the experimental setup, an RF mixer is used. A pulse generator is used to generate a clock signal, as illustrated in Figure S 2a. This is multiplied with any arbitrary RF input signal, say a sine wave, as illustrated in Figure S 2 b. The clock signals get modulated with the input signal as shown in Figure S 2 c. This modulated signal is applied to the AOM, and thus, the femtosecond laser can be both amplitudes modulated, and the sampling frequency can be controlled Figure S 2 d.


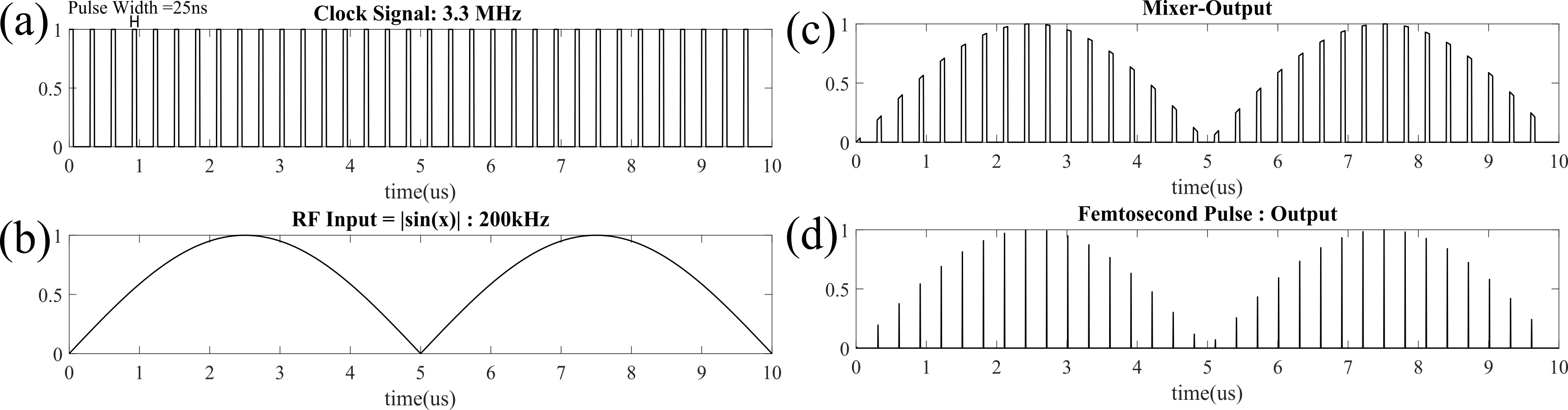


Figure S 2 : A clock signal with pulse width 25 ns and Frequency 3.3 MHz is used to down sample the femtosecond pulse train from 40 MHz to 3.33 MHz. (b) RF signal, |sin(x)| of 200 kHz is used as the RF-input signal, (c) Output of the RF mixer obtained that is sent to the AOM to modulate femtosecond laser pulses. (d) Down sampled and amplitude-modulated femtosecond pulses obtained that is used to switch Sb.

# Frequency Response


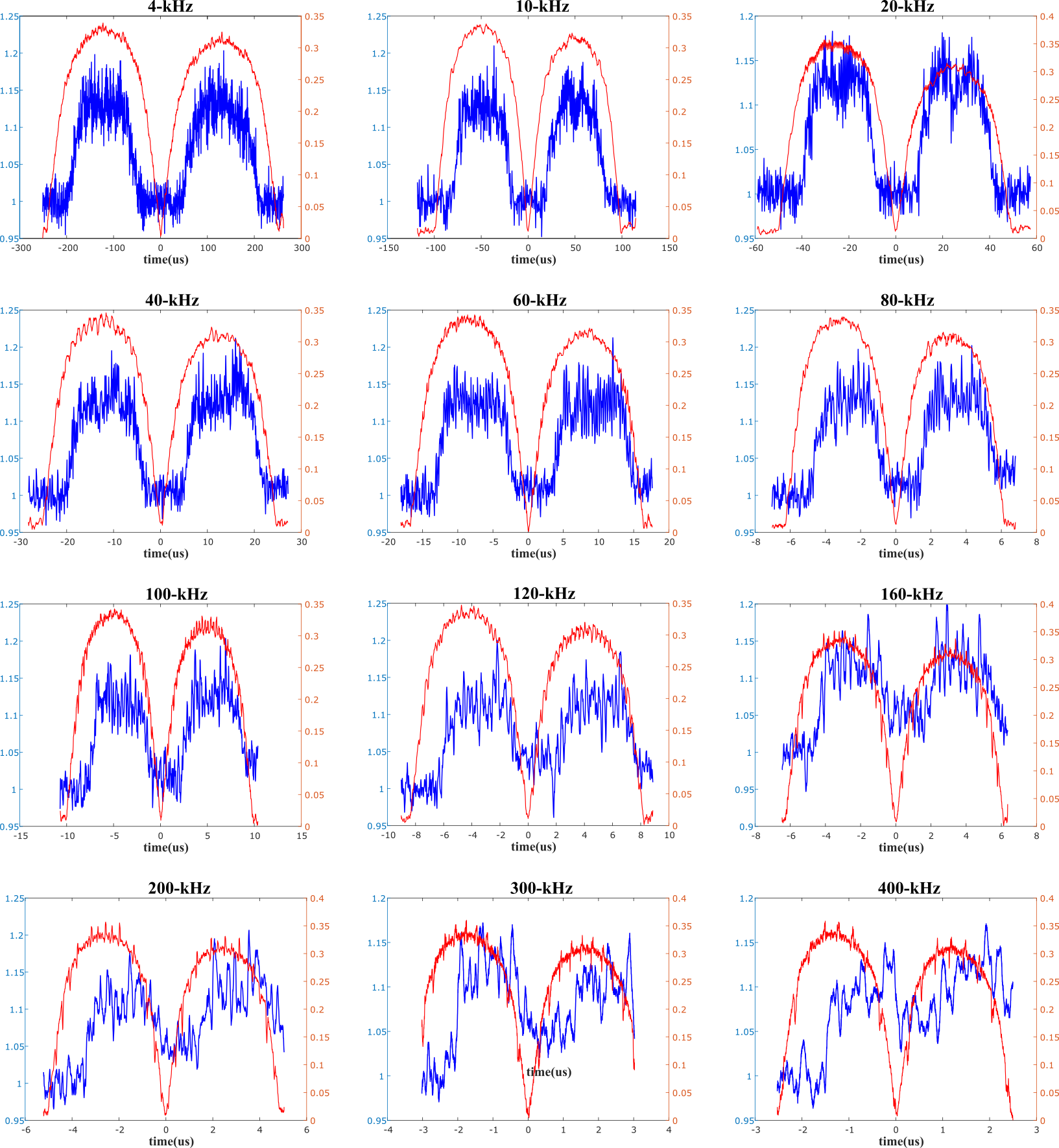


Figure S 3: Sine wave responses of Sb filter cell (in blue) for various input sine wave (in red) of frequencies ranging from 4 kHz-400 kHz.

# Effect of waveguide Crosstalk

Our MWP filter design we use a high energy pump laser to switch Sb on top of a waveguide crossing. Our waveguide crossing design is based on the crossing design demonstrated in [1] , with waveguide crosstalk of -50 dB. Switching of Sb results in modulation of the transmission of the waveguide crossing for probe line (also for pump line) which is recorded in the photodetector using a CW laser. Due to waveguide crossing cross talk, stray signals from high energy pump laser can be recorded onto the photodetectors, causing jitter noise.

It is important to note both pump and probe lasers have different operating wavelengths, thus an optical filter can be further used to decrease the effect of waveguide crossing crosstalk, while monitoring the change in transmission.

# References

# [1] M. Johnson, M. Johnson, M. G. Thompson, and D. Sahin, “Low-loss, low-crosstalk waveguide crossing for scalable integrated silicon photonics applications,” *Optics Express, Vol. 28, Issue 9, pp. 12498-12507*, vol. 28, no. 9, pp. 12498–12507, Apr. 2020, doi: 10.1364/OE.381304.
